# Clinical Outcomes of Patients With Bethesda III or IV Cytology on Fine Needle Aspiration of Thyroid Nodules—A Retrospective Study

**DOI:** 10.1002/edm2.70076

**Published:** 2025-08-02

**Authors:** Adeel Ahmad Khan, Noor Khalil Ebrahim Jasim, Najlaa Essa A. H. Al‐Mannai, Fateen Ata, Rajen Goyal, Tania Jaber

**Affiliations:** ^1^ Cleveland Clinic Akron General Department of Internal Medicine Akron Ohio USA; ^2^ Department of Internal Medicine Hamad Medical Corporation Doha Qatar; ^3^ Department of Endocrinology Hamad Medical Corporation Doha Qatar; ^4^ Department of Pathology Hamad Medical Corporation Doha Qatar; ^5^ Assistant Professor of Clinical Medicine Weill Cornell Medicine Doha Qatar

**Keywords:** Bethesda III, Bethesda IV, fine needle aspiration, indeterminate cytology, thyroid cancer, thyroid nodules

## Abstract

**Introduction:**

The appropriate management strategy for patients with thyroid nodules and indeterminate cytology on fine needle aspiration (FNA) remains unclear, especially in centres where molecular testing is not available. In this retrospective study, we aimed to identify factors predicting the risk of malignancy in these patients.

**Materials and Methods:**

This retrospective study included consecutive patients with thyroid nodules with Bethesda III/IV cytology who underwent surgical management at Hamad Medical Corporation, Qatar, between 01/01/2015 and 30/08/2023. Patients who did not undergo surgical management were excluded. We performed univariate and multivariate logistic regression analysis to assess the factors predicting the risk of malignancy in this population.

**Results:**

Of 449 patients included in the study, the majority were females (72.2%). The mean (SD) age was 43.7 ± 10.7 years. Arab was the most common ethnicity (56.6%), followed by South‐Asian (18.9%) and South‐East Asian (17.8%). Sonographic features of thyroid nodules were classified as ATA very low in 0.9%, low‐risk in 49.1%, intermediate‐risk in 42.05% and high‐risk in 7.95%. 86.2% had Bethesda III cytology and 13.8% had Bethesda IV cytology. Histopathology of thyroidectomy specimens confirmed malignancy in 179 (39.9%) patients. The malignancy rate in Bethesda III was 37.9%, while in Bethesda IV it was 51.6%. In multivariate logistic regression analysis, ATA intermediate (OR of 1.57 (1.03–2.4); *p* = 0.03) and high risk (OR of 3.92 (1.81–8.48); *p* = 0.001) sonographic patterns were predictive of malignancy.

**Conclusion:**

In patients with indeterminate thyroid nodule cytology and in the absence of molecular markers, the ATA sonographic pattern of thyroid nodules can guide decision‐ making for surgical management vs. surveillance.

## Graphical abstract

In patients with indeterminate thyroid nodule cytology on FNA and in the absence of molecular markers, the ATA sonographic pattern of thyroid nodules can guide the decision for surgical management vs. surveillance. ATA intermediate (OR of 1.57 (1.03–2.4); *p* = 0.03) and high risk (OR of 3.92 (1.81–8.48); *p* = 0.001) sonographic patterns are predictive of malignancy in these cases.

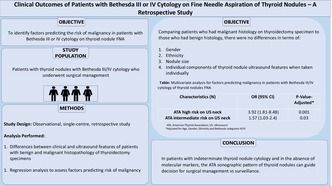

## 1 Introduction

Thyroid nodules are a common clinical condition, and their prevalence depends on the population studied and the methods used to assess thyroid nodules. Studies have shown a prevalence of 19%–35% with ultrasound and 8%–65% in patients with autopsy data. The risk factors for thyroid nodules include advanced age, female gender, and iodine deficiency [1]. Management of thyroid nodules depends on ultrasound (US) features of the nodules, including size, margins, echogenicity, solid components, and presence or absence of high‐risk features such as microcalcifications, taller than wide shape, irregular margins, extra‐thyroidal extension, and presence of suspicious cervical lymph nodes [2]. Based on these features, thyroid nodules can be characterised as having benign, very low suspicion, low suspicion, intermediate suspicion, or high suspicion sonographic pattern. These patterns guide size cut‐off for fine‐needle aspiration (FNA) biopsy [2].

FNA cytology is the most commonly used modality to assess thyroid nodules. The sensitivity of thyroid FNA cytology ranges from 65% to 98%, and specificity ranges from 72% to 100% in the assessment of thyroid nodules, with a false positive rate for detection of thyroid cancer between 0% and 7% and a false negative rate between 1% and 11% based on available literature [3]. The addition of cytopathology has greatly reduced the number of unnecessary surgeries, especially with the advent of a standardised reporting system such as the Bethesda classification system. This system divides cytology results into six categories: I. Non‐diagnostic II. Benign, III. Atypia of undetermined significance (AUS), IV. Follicular neoplasm, V. Suspicious for malignancy, and VI. Malignant, and assigns a risk of malignancy for each category [4].

Best management strategies for patients with indeterminate cytology results on thyroid FNA (Bethesda III/IV) remain controversial. The risk of malignancy in patients with Bethesda III ranges from 13% to 30%, and in Bethesda IV ranges from 23% to 34% [4]. Hence, a significant number of these patients can have benign nodules, and better diagnostic accuracy is needed within this subset to further reduce unnecessary surgeries. Any treatment modality suggested for the management of such cases should take this into account. The American Thyroid Association (ATA) recommends three possible options for managing such cases: surveillance with repeated ultrasonography ± repeat biopsy, diagnostic hemithyroidectomy, or molecular testing [5].

Qatar is a country with a significant number of patients with thyroid malignancy. From 2015 to 2019, 549 new cases of thyroid malignancy were diagnosed in Qatar, contributing significantly to healthcare costs [6]. Molecular studies on cytology specimens are not currently available in our institution or within the country, so patients with indeterminate cytology either undergo a repeat FNA or proceed to surgery. A percentage of these patients then have to undergo a completion thyroidectomy depending on the final pathology. Apart from financial toxicity, this approach also increases the number of unnecessary surgeries and potential complications [7]. It has also been previously well reported that patients' quality of life may be severely affected in patients who have undergone total thyroidectomy [8, 9]. In this retrospective study, we aimed to assess the clinical characteristics and outcomes of patients with Bethesda III or IV diagnostic category on thyroid nodule cytology.

## 2 Materials and Methodology

In this retrospective study, we included consecutive patients who had Bethesda III/IV cytology on FNA of thyroid nodules done at Hamad Medical Corporation, Qatar, between 01/01/2015 and 30/08/2023 and who were surgically managed. All patients had neck ultrasonography in our institution. Patients under 14 years and those with FNA cytology other than Bethesda III/IV or who did not have follow up were excluded from the study. Neck ultrasound images were reviewed by a radiologist and thyroidologist to classify nodules according to the ATA sonographic pattern.

Demographic data was collected from the electronic medical record (Cerner) and included demographic details, ultrasound characteristics of thyroid nodules, number of FNAs performed, type of surgical management, and histopathology of surgically removed thyroid specimens. Cytology and ultrasound reports were correlated with the pathology reports.

We used descriptive statistics to present the demographic data of the study cohort. We used mean (SD) and median (IQR) to summarise continuous variables, while categorical variables were summarised as percentages. We performed univariate and multivariate logistic regression analysis to assess the factors predicting the risk of malignancy in thyroid nodules with Bethesda III or IV category on FNA cytology. A *p*‐value of < 0.05 was considered significant. We used STATA 18 for the analysis.

## 3 Results

Table 1 highlights the baseline and demographic characteristics of the study population. A total of 449 patients were included in the study, the majority of whom were females (*N* = 324, 72.2%). The mean (SD) age was 43.7 ± 10.7 years. Arab was the most common ethnicity (*N* = 254, 56.6%) followed by South Asian (*N* = 85, 18.9%) and South‐East Asian (*N* = 80, 17.8%). Based on ultrasound characteristics, most of the patients had thyroid nodules between > 2 and ≤ 4 cm in size (*N* = 191, 43.2%), hypoechoic (*N* = 204, 48.1%), solid (*N* = 331, 74.7%) and had regular margins (*N* = 373, 90.5%). Most (*N* = 300, 73%) of the patients did not have any calcifications on US, whereas microcalcification was noted in 69 (16.8%) patients. US evidence of suspicious lymph nodes was reported only in 8 (1.9%) patients. The majority of the patients had thyroid nodules categorised as ATA very low risk (4, 0.9%), low‐risk for malignancy (216, 49.1%), followed by intermediate‐risk (*N* = 185, 42.05%) and high‐risk (*N* = 35, 7.95%). 387 (86.2%) patients had Bethesda III and 62 (13.8%) had Bethesda IV cytology results.

**TABLE 1:** Baseline characteristics of patients with Bethesda III/IV cytology on thyroid nodules FNA.

Characteristic	Units	Result
Total Patients	*N*	449
Age, Mean ± SD	Years	43.7 ± 10.7
Gender		
Female	*N* (%)	324 (72.2)
Male		125 (27.8)
Ethnicity		
South Asian	*N* (%)	85 (18.9)
South‐East Asian		80 (17.8)
Arabs		254 (56.6)
Qatari		77 (17.1)
Other Arabs		177 (39.4)
Others		30 (6.7)
Nodule size (cm) (*N* = 442)		
≤ 1	*N* (%)	28 (6.3)
> 1 to ≤ 2		92 (20.8)
> 2 to ≤ 4		191 (43.2)
> 4		131 (29.6)
Nodule echogenicity (*N* = 422)		
Hyperechoic	*N* (%)	26 (6.2)
Hypoechoic		204 (48.3)
Isoechoic		177 (41.9)
Heterogenous		15 (3.5)
Nodule consistency (*N* = 443)		
Solid	*N* (%)	331 (74.7)
Cystic		19 (4.3)
Solid‐cystic		92 (20.8)
Spongiform		1 (0.2)
Clacification (*N* = 411)		
Micro	*N* (%)	69 (16.8)
Macro		33 (8)
Peripheral		9 (2.2)
None		300 (73)
Nodule margins (*N* = 412)		
Regular	*N* (%)	373 (90.5)
Irregular		31 (7.5)
Lobulated		8 (1.9)
Lymph nodes (*N* = 421)		
Benign	*N* (%)	154 (36.6)
Suspicious		8 (1.9)
None		259 (61.5)
ATA risk of malignancy (*N* = 440)		
Very low	*N* (%)	4 (0.9)
Low		216 (49.1)
Intermediate		185 (42.05)
High		35 (7.95)
Bethesda classification (*N* = 449)		
III	*N* (%)	387 (86.2)
IV		62 (13.8)
Cytopathology (*N* = 449)		
AUS	*N* (%)	72 (16)
FLUS		316 (70.4)
Follicular Neoplasm		40 (8.9)
Suspicious for Follicular neoplasm		21 (4.7)
Total number of FNA done (*N* = 449)		
1	*N* (%)	312 (69.5)
2		11 (24.7)
3		22 (4.9)
4		3 (0.7)
6		1 (0.2)

Abbreviations: AUS, atypia of undetermined significance; FLUS, follicular lesion of undetermined significance; FNA, fine needle aspiration.

**FIGURE 1:**
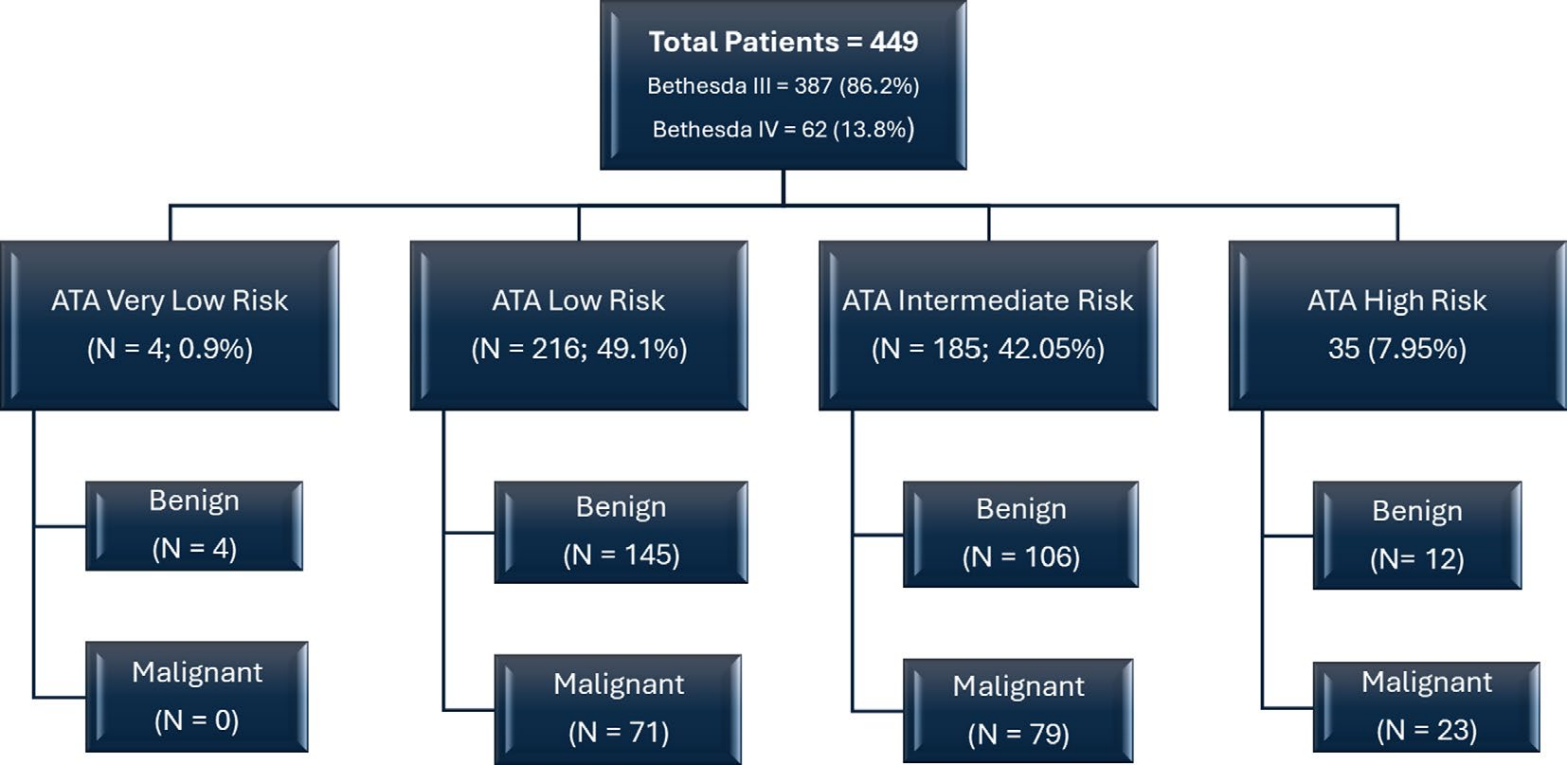
Histopathology results based on ATA risk categories.

Table 2 highlights the management of the study group. Around 30% of patients had repeat FNA biopsy prior to proceeding with surgery. Hemithyroidectomy alone was the most common surgical intervention performed (*N* = 264, 58.8%), whereas total thyroidectomy was performed in 154 (34.3%) patients. 31 (6.9%) patients underwent a completion thyroidectomy following initial hemithyroidectomy. Malignancy was reported in 179 (39.9%) patients on final histopathology specimen whereas 270 (60.1%) had benign histopathology (NIFTP have been categorised as benign). Classic variant PTC was the most common thyroid malignancy (*N* = 79, 44.1%). Malignancy rate in Bethesda III was 37.9% (*N* = 147/387) and 51.6% (*N* = 32/62) in Bethesda IV. Figure 1 shows the proportion of benign and malignant histopathologies based on ATA risk categories.

**TABLE 2:** Management and outcomes of patients with Bethesda III/IV on thyroid nodules FNA.

Characteristic	Units	Result
Surgical management (*N* = 449)		
Hemithyroidectomy alone	*N* (%)	264 (58.8)
Hemithyroidectomy followed by completion thyroidectomy		31 (6.9)
Total thyroidectomy		154 (34.3)
Surgical histopathology (*N* = 449)		
Benign	*N* (%)	270 (60.1)
Benign nodular disease		234 (86.7)
Chronic Lymphocytic thyroiditis		22 (8.1)
NIFTP		13 (4.8)
Riedel's thyroiditis		1 (0.4)
Malignant		179 (39.9)
PTC		146 (81.6)
PTC, classic variant		79 (44.1)
Micro PTC		38 (21.2)
PTC, Follicular variant		24 (13.4)
PTC, oncocytic variant		3 (1.7)
PTC, tall cell variant		2 (1.1)
FTC		28 (15.6)
Mixed PTC and FTC		2 (1.1)
MTC		1 (0.6)
Hurthle cell carcinoma		1 (0.6)
Mixed PTC and Hurthle cell carcinoma		1 (0.6)
TNM staging (*N* = 173)		
T1a	*N* (%)	70 (40.5)
T1b		30 (17.3)
T2		34 (19.6)
T3a		37 (21.4)
T3b		2 (1.2)
N1a		4 (2.3)
N1b		5 (2.9)
N0/Nx		164 (94.8)
M1		1 (0.6)

Abbreviations: FTC, follicular thyroid carcinoma; MTC, medullary thyroid cancer; NIFTP, non‐invasive follicular thyroid neoplasm with papillary‐like nuclear features; PTC, papillary thyroid carcinoma.

Patients with malignant histopathology on thyroidectomy specimens were more likely to have an ATA intermediate (45.7% vs. 39.7%; *p* = 0.001) and high risk (13.3% vs. 4.5%; *p* = 0.001) sonographic pattern based on US features. They were also more likely to have Bethesda IV on FNA cytology (17.9% vs. 11.1%; *p* = 0.04) compared to those with benign histopathology (Table 3).

**TABLE 3:** Differences between clinical and ultrasound features of patients with benign and malignant histopathology of thyroidectomy specimen.

Variable	Units	Benign (*N* = 270)	Malignant (*N* = 179)	*p*
Age, Mean ± SD	Years	43.3 ± 10.7	44.2 ± 10.5	0.38
Gender				
Female	*N* (%)	199 (73.7)	125 (69.8)	0.37
Male		71 (26.3)	54 (30.2)	
Ethnicity				
South Asian	*N* (%)	48 (17.8)	37 (20.7)	0.47
South‐East Asian		44 (16.3)	36 (20.1)	
Qatari		44 (16.3)	33 (18.4)	
Other Arabs		114 (42.2)	63 (35.2)	
Others		20 (7.4)	10 (5.6)	
Nodule size (cm) (*N* = 442)				
≤ 1	*N* (%)	18 (6.8)	10 (5.6)	0.26
> 1 to ≤ 2		47 (17.8)	45 (25.4)	
> 2 to ≤ 4		120 (45.3)	71 (40.1)	
> 4		80 (30.2)	51 (28.8)	
Nodule echogenicity (*N* = 422)				
Hyperechoic	*N* (%)	16 (2.75)	10 (5.9)	0.1
Hypoechoic		112 (43.9)	92 (54.4)	
Isoechoic		119 (46.7)	58 (34.3)	
Heterogenous		7 (2.7)	8 (4.7)	
Nodule consistency (*N* = 443)				
Solid	*N* (%)	194 (72.4)	137 (78.3)	0.38
Cystic		11 (4.1)	8 (4.6)	
Solid‐cystic		63 (23.1)	30 (17.1)	
Spongiform		1 (0.4)	0	
Calcification (*N* = 411)				
Micro	*N* (%)	33 (13.1)	35 (22)	0.06
Macro		21 (8.3)	12 (7.5)	
Peripheral		4 (1.6)	5 (3.1)	
None		194 (77)	106 (66.7)	
Nodule margins (*N* = 412)				
Regular	*N* (%)	229 (92.7)	144 (87.3)	0.15
Irregular		15 (6.1)	16 (9.7)	
Lobulated		3 (1.2)	5 (3)	
Lymph nodes (*N* = 421)				
Benign	*N* (%)	87 (34.4)	67 (39.8)	0.051
Suspicious		2 (0.8)	6 (3.6)	
None		164 (64.8)	95 (56.5)	
ATA risk of malignancy (*N* = 440)				
Very low	*N* (%)	4 (1.5)	0	0.001
Low		145 (54.3)	71 (41)	
Intermediate		106 (39.7)	79 (45.7)	
High		12 (4.5)	23 (13.3)	
Bethesda classification (*N* = 449)				
III	*N* (%)	240 (88.9)	147 (82.1)	0.04
IV		30 (11.1)	32 (17.9)	

In univariate logistic regression analysis, only the presence of microcalcifications (OR of 1.94 (1.14–3.3); *p* = 0.01), ATA intermediate risk (OR of 1.52 (1.01–2.28), *p* = 0.04), ATA high risk (OR of 3.9 (1.84–8.3); *p* < 0.001) and Bethesda IV on FNA cytology (OR of 1.74 (1.01–2.98); *p* = 0.04) were associated with malignancy (Table 4). There was no association between different ethnicities and the risk of malignancy in the univariate analysis (Table 4). In multivariate logistic regression analysis, ATA intermediate (OR of 1.57 (1.03–2.4); *p* = 0.03) and high risk (OR of 3.92 (1.81–8.48); *p* = 0.001) sonographic patterns were predictive of malignancy after adjusting for age, gender, ethnicity, and Bethesda categories (Table 5).

**TABLE 4:** Univariate analysis for factors predicting malignancy in patients with Bethesda III/IV cytology of thyroid nodules FNA.

Characteristics (*N*)	OR (95% CI)	*p*
Age	1.007 (0.99–1.02)	0.38
Male gender	1.21 (0.79–1.84)	0.37
Ethnicity		
South‐East Asian	1.48 (0.86–2.53)	0.15
South‐Asian	1.39 (0.82–2.36)	0.22
Qatari	1.35 (0.78–2.34)	0.27
Others	0.9 (0.39–2.05)	0.8
Nodule consistency		
Solid	0.97 (0.38–2.47)	0.95
Solid‐cystic	0.66 (0.24–1.82)	0.43
Nodule margins		
Irregular	1.69 (0.81–3.53)	0.16
Lobulated	2.65 (0.62–11.25)	0.18
Nodule echogenicity		
Hypoechoic	1.31 (0.57–3.03)	0.5
Isoechoic	0.78 (0.33–1.82)	0.57
Heterogenous	1.82 (0.51–6.61)	0.36
Nodule size		
> 4 cm	1.14 (0.49–2.68)	0.75
> 2 to ≤ 4 cm	1.06 (0.46–2.43)	0.88
> 1 to ≤ 2 cm	1.72 (0.72–4.13)	0.22
Nodule calcification		
Microcalcification	1.94 (1.14–3.3)	0.01
Macrocalcification	1.04 (0.49–2.2)	0.9
Lymph node on US neck		
Suspicious for malignancy	3.89 (0.76–19.9)	0.1
None	0.75 (0.5–1.12)	0.17
ATA risk of malignancy		
ATA high risk on US neck	3.9 (1.84–8.3)	< 0.001
ATA intermediate risk on US neck	1.52 (1.01–2.28)	0.04

**TABLE 5:** Multivariate analysis for factors predicting malignancy in patients with Bethesda III/IV cytology of thyroid nodules FNA.

Characteristics (*N*)	OR (95% CI)	*p* Adjusted^a^
ATA high risk on US neck	3.92 (1.81–8.48)	0.001
ATA intermediate risk on US neck	1.57 (1.03–2.4)	0.03

Abbreviations: ATA, American Thyroid Association; US, ultrasound.

^a^ Adjusted for age, gender, ethnicity and Bethesda categories III/IV.

## 4 Discussion

In this retrospective study of 449 patients, we analysed the risk of malignancy and the factors predicting this risk in one of the largest cohorts of patients with thyroid nodules with indeterminate cytology on thyroid FNA who underwent thyroidectomy. To our knowledge, this is the first and largest study in this region to do so. The majority of patients had thyroid nodules with low risk for malignancy ATA sonographic pattern, followed by intermediate risk and high risk. The rate of malignancy in this cohort was 39.9% with classic variant PTC being the most prevalent thyroid cancer subtype. In multivariate logistic regression analysis, ATA intermediate and high risk sonographic patterns were predictive of malignancy.

A major strength of this study is that the estimate of the risk of malignancy in indeterminate thyroid nodules in this study may be more accurate than previously reported, as all patients in this study underwent surgery. Having said that, we are well aware that the heterogeneity in reporting AUS cytology and the subsequent rate of malignancy in indeterminate nodules varies among institutions, which explains the variable literature.

The rate of malignancy in our cohort is considerably higher than that reported by Ali et al., underscoring the importance of conducting region‐specific and institution‐specific studies [4]. Our results are consistent with a study by Alyusuf et al., who, in a cohort of 278 patients with Bethesda III and IV thyroid nodules, showed a 39.9% malignancy rate [10]. In another study by Ho et al. consisting of 381 surgically managed patients with Bethesda III FNA cytology, a malignancy rate of 37.8% was noted, similar to our cohort [11]. On the other hand, a study from the subcontinent showed a much lower malignancy rate in patients with Bethesda III compared to our cohort (29.6% vs. 37.9%) and a similar rate in Bethesda IV patients (47.1% vs. 56.1%) [12]. However, the study cohort was very small and included only 27 patients in Bethesda III and 87 patients in Bethesda IV. Mosca et al. reported a malignancy rate of only 12% (*N* = 384) in Bethesda III and 11.2% (*N* = 242) in Bethesda IV patients, much lower than our cohort [13]. This could be related to demographic differences between the two cohorts. A significant proportion of our patients consisted of South Asian and South East Asian origin (*N* = 165, 36.74%), who are known to have a higher incidence of thyroid malignancy compared to other ethnicities [14, 15, 16].

The variable and heterogeneous results reported in the literature further underscore the need for better diagnostic accuracy for this subset of nodules. Recently, Latia et al. found stiffness on elastography as an indicator of malignancy in patients with Bethesda IV thyroid nodules [17]. Stoian et al. report a sensitivity of 89.5% for detecting thyroid malignancy using combined ultrasound and elastography in patients with Bethesda III nodules [18]. Elastography and contrast‐enhanced ultrasound remain exploratory in their role in distinguishing malignancy in thyroid nodules. While their combined use may be a useful non‐diagnostic adjunct to conventional US, they are yet to be validated as compared to ATA risk patterns [19]. Molecular assays have recently emerged as a diagnostic tool that may offer improved diagnostic accuracy and decrease unnecessary surgeries. In the absence of these markers, however, management of patients with thyroid nodules with indeterminate FNA cytology poses a significant challenge. Several studies have attempted to identify factors that can help in the decision‐making process. Alyusuf et al. concluded that the presence of hypoechogenicity and calcifications on thyroid US was independently associated with two‐fold increased risk of malignancy in patients with indeterminate FNA cytology [10]. In a meta‐analysis, Gao et al. concluded that among patients with Bethesda III thyroid nodules, the presence of one high‐risk US feature (hypoechogenicity, calcifications, irregular margin, taller‐than‐wide shape, or increase in nodule size during follow‐up) had a 75% sensitivity of predicting underlying malignancy. Moreover, the likelihood of malignancy increased as the number of suspicious features on US increased [20]. Similarly, a meta‐analysis performed by Li et al. found that irregular nodule margins, solitary nodules, hypoechogenicity, microcalcifications, and being taller than wide are independently associated with underlying malignancy in thyroid nodules [21]. Similar associations between different sonographic features and thyroid malignancy have been reported in several other studies [22, 23]. On the other hand, Sahin et al. did not find any significant association between sonographic characteristics of thyroid nodules and the risk of malignancy [24]. In our study, except for the presence of microcalcifications, none of the other sonographic features, if taken alone, were predictive of malignancy in thyroid nodules. However, when all the sonographic features were interpreted together as ATA risk sonographic pattern, these features collectively predicted malignancy, as evidenced by the association between ATA intermediate and high‐risk categories and malignancy in our cohort (Tables 4 and 5). This is again in accordance with the latest published guidelines by ATA [4].

Our study has several strengths. With a sample size of 449 patients, this is the largest cohort for predicting malignancy in indeterminate thyroid nodules. Another important strength of our study is the inclusion of a multi‐ethnic population consisting of South Asians, Southeast Asians, and Arabs, thus enabling a deeper understanding of the clinical implications of indeterminate thyroid nodules in this previously understudied group of patients. Moreover, to our knowledge, this is the first study in the region validating the utility of ATA risk categorisation in the assessment of patients with indeterminate thyroid nodules. However, the study is retrospective, which means that the effect of potential confounders cannot be eliminated. Specifically, since the study included only patients with surgically managed Bethesda III and IV nodules, the risk of selection bias needs to be kept in mind while interpreting the results. Such limitations are inherent to the retrospective designs and can only be addressed via prospective studies to follow the outcomes of patients with indeterminate cytology who did not undergo surgery and compare them to those who underwent surgery. Another limitation of this study is the lack of exploration of the impact of non‐invasive follicular thyroid cancer with papillary‐like features (NIFTP) on malignancy rate. Since the sample size of NIFTP patients in our study was very small and the study did not include any prolonged follow‐up of patients diagnosed with NIFTP, the potential impact of NIFTP on malignancy rates in our study could not be calculated.

## 5 Conclusion

In patients with thyroid nodules with indeterminate cytology and in the absence of molecular assays, clinicians can rely on the ATA sonographic pattern of thyroid nodules and risk of malignancy to help guide the need for surgery. In a low risk pattern, surveillance with neck ultrasonography may be sufficient.

## Author Contributions

**Adeel Ahmad Khan:** conceptualisation, data curation, formal analysis, investigation, methodology, validation, visualisation, writing – original draft, writing – review and editing. **Noor Khalil Ebrahim Jasim**, **Najlaa Essa A. H. Al‐Mannai:** data curation, writing – original draft. **Fateen Ata:** formal analysis, investigation, visualisation, writing – original draft. **Rajen Goyal:** data curation. **Tania Jaber:** principal investigator, validation, conceptualisation, supervision, writing – review and editing.

## Disclosure

The authors have nothing to report.

## Ethics Statement

The study has been approved by the Medical Research Centre (MRC) at Hamad Medical Corporation, Qatar, under the approval ID MRC‐01‐23‐798. The research was conducted in compliance with the ethical principles outlined in the Declaration of Helsinki. Given the retrospective nature of this study and the de‐identified patient data, a waiver of informed consent was obtained from the MRC at Hamad Medical Corporation. We anonymised all data prior to analysis by removing personally identifiable information and assigning unique codes to each participant. All data were handled following institutional and ethical guidelines.

## Conflicts of Interest

The authors declare no conflicts of interest.

## Data Availability

The data that support the findings of this study are available from the corresponding author upon reasonable request.
