# Correction to ‘Clinical Outcomes of Patients With Bethesda III or IV Cytology on Fine Needle Aspiration of Thyroid Nodules—A Retrospective Study’

**DOI:** 10.1002/edm2.70106

**Published:** 2025-09-12

Khan AA, Jasim NKE, Al‐Mannai NEAH, Ata F, Goyal R, Jaber T. Clinical Outcomes of Patients With Bethesda III or IV Cytology on Fine Needle Aspiration of Thyroid Nodules‐A Retrospective Study. Endocrinol Diabetes Metab. 2025;8(5):e70076.

In the Acknowledgements section on Page 7 of the manuscript, the APC for publication was attributed to the wrong entity.

Instead of ‘Article processing charges for the publication of this study were provided by the Qatar National Library (QNL)’, we would like to change this to:

Article processing charges for the publication of this study were provided by the Medical Research Center (MRC) at Hamad Medical Corporation (HMC).

We apologise for this error.

